# Telerehabilitation vs. centre-based cardiac rehabilitation after acute coronary syndrome: results of the pragmatic TELECOR randomized trial

**DOI:** 10.1093/ehjopen/oeag142

**Published:** 2026-09-24

**Authors:** Maria Boldó Alcaine, Maria José Durà Mata, Gabriel-Fernando Sandoval Ruíz, Maria Llaberia Marcual, Marta Carceller de la Torre, Mar Domingo Teixidor, Evelyn Santiago Vacas, Antoni Bayés Genís

**Affiliations:** Physical Medicine and Rehabilitation Department, University Hospital Germans Trias i Pujol, Carretera del Canyet, s/n, 08916 Badalona, Barcelona, Spain; Heart Failure Clinic, University Hospital Germans Trias I Pujol, Carretera del Canyet, s/n, 08916 Badalona, Barcelona, Spain; Department of Medicine, Universitat Autònoma de Barcelona, Pg. Vall d’Hebron, 119–129, Edifici Pavelló Docent, 08023 Barcelona, Spain; Physical Medicine and Rehabilitation Department, University Hospital Germans Trias i Pujol, Carretera del Canyet, s/n, 08916 Badalona, Barcelona, Spain; Department of Medicine, Universitat Autònoma de Barcelona, Pg. Vall d’Hebron, 119–129, Edifici Pavelló Docent, 08023 Barcelona, Spain; Physical Medicine and Rehabilitation Department, University Hospital Germans Trias i Pujol, Carretera del Canyet, s/n, 08916 Badalona, Barcelona, Spain; Physical Medicine and Rehabilitation Department, University Hospital Germans Trias i Pujol, Carretera del Canyet, s/n, 08916 Badalona, Barcelona, Spain; Physical Medicine and Rehabilitation Department, University Hospital Germans Trias i Pujol, Carretera del Canyet, s/n, 08916 Badalona, Barcelona, Spain; Heart Failure Clinic, University Hospital Germans Trias I Pujol, Carretera del Canyet, s/n, 08916 Badalona, Barcelona, Spain; Cardiology Department, University Hospital Germans Trias I Pujol, Carretera del Canyet, s/n, 08916 Badalona, Barcelona, Spain; CIBER Cardiovascular (CIBERCV), Instituto de Salud Carlos III, Avenida Monforte de Lemos, 3–5, Pabellón 11, 28029 Madrid, Spain; Heart Failure Clinic, University Hospital Germans Trias I Pujol, Carretera del Canyet, s/n, 08916 Badalona, Barcelona, Spain; Cardiology Department, University Hospital Germans Trias I Pujol, Carretera del Canyet, s/n, 08916 Badalona, Barcelona, Spain; CIBER Cardiovascular (CIBERCV), Instituto de Salud Carlos III, Avenida Monforte de Lemos, 3–5, Pabellón 11, 28029 Madrid, Spain; Heart Failure Clinic, University Hospital Germans Trias I Pujol, Carretera del Canyet, s/n, 08916 Badalona, Barcelona, Spain; Department of Medicine, Universitat Autònoma de Barcelona, Pg. Vall d’Hebron, 119–129, Edifici Pavelló Docent, 08023 Barcelona, Spain; Cardiology Department, University Hospital Germans Trias I Pujol, Carretera del Canyet, s/n, 08916 Badalona, Barcelona, Spain; CIBER Cardiovascular (CIBERCV), Instituto de Salud Carlos III, Avenida Monforte de Lemos, 3–5, Pabellón 11, 28029 Madrid, Spain

**Keywords:** Cardiac rehabilitation, Acute coronary syndrome, Telerehabilitation, Secondary prevention, Exercise therapy, Digital health

## Abstract

**Aims:**

Cardiac rehabilitation (CR) improves cardiovascular outcomes after acute coronary syndrome (ACS), but access remains suboptimal. Telerehabilitation may overcome barriers, yet evidence from real-world, public health systems is limited. This randomized controlled trial study aimed to evaluate the effectiveness of a structured telerehabilitation programme compared with standard centre-based CR in patients after ACS, focussing on cardiovascular risk factor control and lifestyle adherence.

**Methods and results:**

TELECOR is a pragmatic, open-label, randomized trial conducted at a tertiary public hospital. Adults with recent ACS were randomized 1:1 to an 8 week structured telerehabilitation or a centre-based CR programme. The primary outcome was achievement of a composite target (≥4 of 7 predefined cardiovascular and lifestyle goals) at 8 weeks. Secondary outcomes included changes in functional capacity, medication adherence, quality of life, psychological status, satisfaction, and cardiovascular events at 6 months. A total of 129 patients were randomized (65 telerehabilitation, 64 centre-based). At 8 weeks, 86% of telerehabilitation vs. 79.7% of centre-based participants met the composite target (*P* = 0.37). At 6 months, this was 87% vs. 80.4% (*P* = 0.34). Both groups showed similar improvements in functional capacity (≈+1 metabolic equivalent of tasks), adherence (>84%), and satisfaction (>91/100). No between-group differences were observed in clinical events or psychological outcomes.

**Conclusion:**

In a real-world public hospital setting, telerehabilitation was non-inferior to conventional CR in achieving cardiovascular risk factor control and improving functional capacity. These results support the broader integration of digital CR models into routine secondary prevention care.

## Graphical Abstract

**Graphical Abstract:**
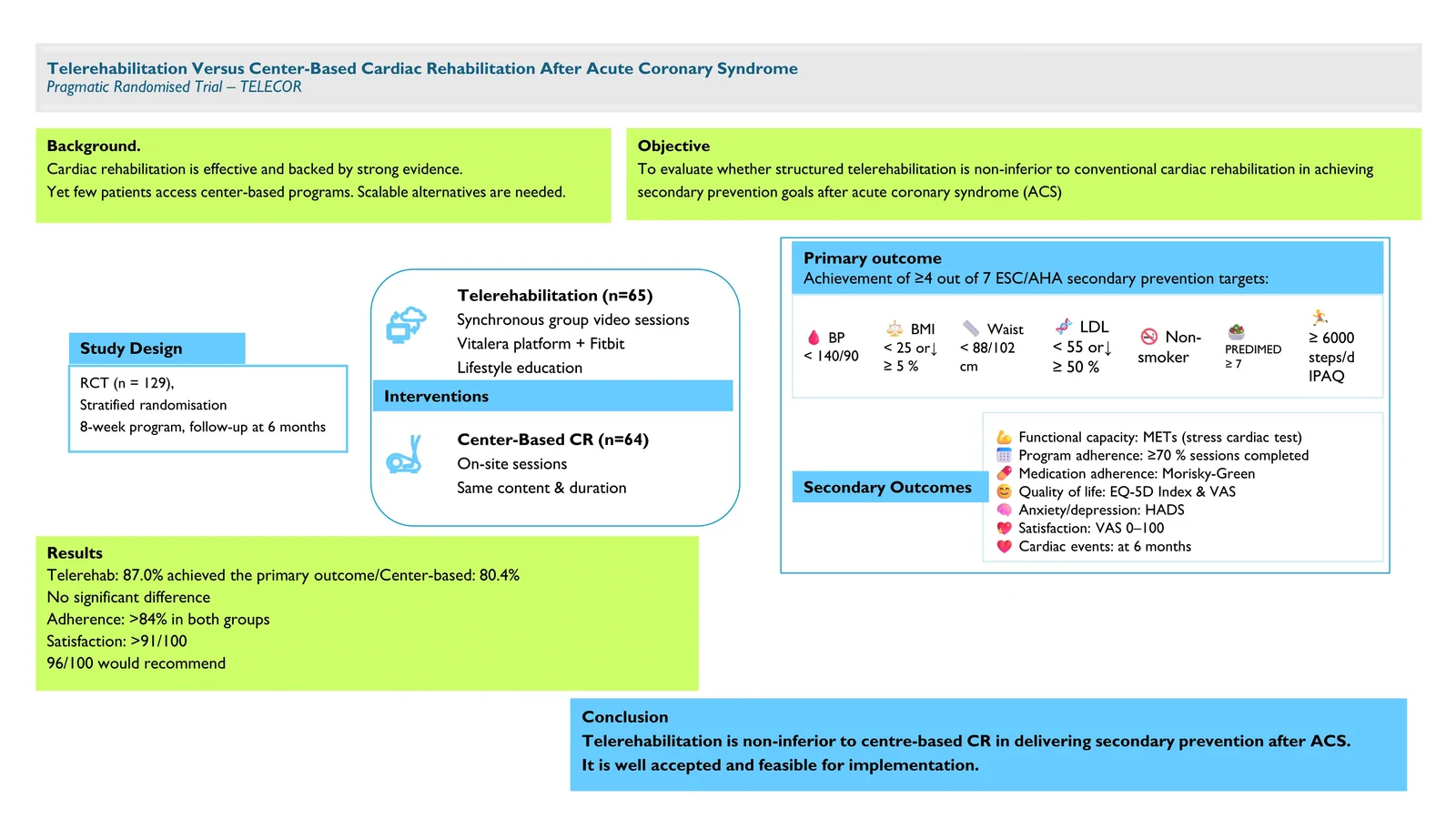

## Introduction

Cardiac rehabilitation (CR) is a cornerstone of secondary prevention in patients with ischaemic heart disease, strongly endorsed by clinical guidelines from the European Society of Cardiology (ESC)^1^ and the American Heart Association (AHA).^2^ CR has been shown to reduce mortality, hospital readmissions, and improve functional recovery and quality of life.^3^ However, despite these well-documented benefits, CR remains substantially underutilized, particularly within publicly funded health systems—due to barriers such as geographic distance, limited programme availability, scheduling conflicts, and patient-level factors.^4,5,6^ Consequently, many eligible patients are not referred, enrolled, or do not complete the programme, limiting its public health impact.

To address these persistent challenges, alternative models such as home-based and telerehabilitation programmes have gained increasing attention. These models aim to deliver structured exercise training, cardiovascular education, and clinical monitoring through digital platforms, offering more flexible and accessible formats that reduce reliance on physical attendance. The 2023 AHA/ACC/ACCP/ASPC/NLA/PCNA guideline on the management of chronic coronary disease recognizes the role of technology-enabled CR and highlights the need for pragmatic research to support its implementation in real-world settings.^2,7^ Meta-analyses and randomized trials indicate that may achieve comparable improvements in physical capacity, adherence, and patient satisfaction compared with centre-based programmes. However, much of this evidence derives from small, highly controlled studies.^8,9^ As a result, important questions remain regarding long-term sustainability, scalability, patient selection, and the integration of telerehabilitation into routine clinical pathways. Questions remain regarding real-world scalability, patient selection, and integration into clinical pathways, particularly within publicly funded healthcare systems.^10,11,12,13^ Furthermore, few trials have assessed cardiovascular risk factor control as a primary outcome using multidimensional composite endpoints aligned with guideline-based targets.

To address these gaps, we developed and implemented TELECOR, a pragmatic, randomized controlled trial designed to evaluate the clinical effectiveness of a structured telerehabilitation programme fully integrated into a tertiary public hospital in Spain. The intervention consists of personalized exercise prescription, digital monitoring, and interactive follow-up via a secure telehealth platform (Vitalera). The primary objective is to compare the proportion of patients achieving a composite goal of cardiovascular risk factor control and healthy lifestyle adherence following acute coronary syndrome (ACS), in both telerehabilitation and standard centre-based CR programmes.

## Methods

### Study design

Patients were randomly assigned (1:1) to telerehabilitation or conventional CR using a computer-generated allocation sequence in blocks of four, stratified by age and sex. The randomization sequence was generated by an independent statistician using dedicated software. Group assignments were placed in sequentially numbered, opaque, sealed envelopes prepared externally and kept confidential.

The complete trial protocol was registered and is publicly available at ClinicalTrials.gov (Identifier: NCT06807658). The study is sponsored by Germans Trias i Pujol Hospital, which also provided the protocol as the responsible party. Participants were enrolled by the principal investigator. Group allocation was determined at the time of enrolment by opening the next envelope in sequence, ensuring full allocation concealment.

### Participants

Patients were recruited consecutively from the population referred to the Cardiac Rehabilitation Unit by the Cardiology Department following ACS; all consecutive patients who met the eligibility criteria were screened and invited to participate. Recruitment took place between October 2021 and January 2023. Eligible participants were adults (>18 years) who had experienced an ACS within the previous 2 months. Inclusion criteria included Killip class I–II, no previous myocardial infarction or angina, no ischaemia or arrhythmia on complementary testing, left ventricular ejection fraction ≥35%, and functional capacity >5 metabolic equivalent of tasks (METs). Patients were excluded if they had physical or cognitive impairments precluding the use of digital technology or if they refused to sign informed consent. Patients with a left ventricular ejection fraction ≤35% or a functional capacity below 5 METs were not eligible, as these characteristics are associated with higher exercise-related cardiovascular risk and generally require supervised centre-based rehabilitation with closer clinical monitoring during the initial phases of recovery. Moderate, non-revascularized coronary lesions, when present, were managed conservatively according to current guideline-recommended clinical practice. All participants were considered clinically stable and suitable for cardiac rehabilitation at enrolment.

### Interventions

Participants in both arms completed a structured 8 week CR programme consisting of 20 sessions focussed on exercise and lifestyle education. Both groups received the same multidisciplinary cardiac rehabilitation programme; only the exercise setting, the modality of aerobic exercise, and the heart-rate monitoring system differed between interventions. The exercise prescription, progression criteria, therapeutic education, and cardiovascular risk factor management were identical in both groups.

- Telerehabilitation group: Received group-based sessions (4–6 patients) delivered synchronously via a secure telehealth platform (Vitalera), supplemented by wearable activity tracking (Fitbit Inspire 2).
- Centre-based group: Attended on-site sessions at the hospital’s outpatient rehabilitation unit with in-person monitoring and supervision

Exercise training included aerobic and resistance components, with one-hour supervised sessions delivered on alternate days. Aerobic exercise was individually prescribed according to the baseline symptom-limited treadmill exercise test (Bruce protocol). In participants who achieved a maximal heart rate during the test, aerobic intensity was prescribed at 75%–85% of the maximum heart rate achieved. When a maximal heart rate was not reached, exercise intensity was prescribed using the heart rate reserve (Karvonen) method. In the centre-based programme, aerobic exercise was performed on treadmills and cycle ergometres under direct supervision. In the telerehabilitation programme, aerobic exercise consisted of a supervised home-based circuit combining marching in place, jogging in place, side stepping, step-touch movements, alternating knee lifts, hamstring curls, low-impact jumping jacks, boxing movements and, when appropriate, stair climbing. Heart rate was monitored throughout all exercise sessions. In the telerehabilitation group, monitoring was performed using Fitbit Inspire 2 devices integrated into the Vitalera platform, whereas in the centre-based rehabilitation group, heart rate was monitored using a custo belt (custo med GmbH, Germany) strap during supervised exercise sessions.

Resistance training targeted the major muscle groups using elastic resistance bands (TheraBand). The programme included biceps curls, triceps extensions, chest press, seated row, shoulder flexion, squats, hip flexion, hip abduction, standing heel raises, and front and side planks. Participants performed 2–3 sets of 10–12 repetitions. The initial TheraBand resistance level (blue, black, silver or gold) was selected according to the baseline 10-repetition maximum (10-RM) assessment and reassessed weekly. Resistance was progressed according to repeated 10-RM assessments, with perceived exertion monitored during each session using the OMNI-Resistance Exercise Scale (OMNI-RES; target 4–6), applying the same progression criteria in both study groups.

A detailed description of the exercise protocol, including exercise prescription, progression criteria, and training components, is provided in Supplementary material online, **Appendix**  ***S1***.

### Outcomes

The primary outcome was the proportion of patients meeting a composite endpoint for cardiovascular risk factor control and healthy lifestyle adherence at 8 weeks. The composite endpoint was based on seven cardiovascular risk factors and lifestyle goals derived from ESC and AHA/ACC guidelines for secondary prevention. The threshold of ≥4 goals was chosen pragmatically, as it reflects partial but clinically meaningful adherence to guideline-based secondary prevention. The composite was considered positive if the patient met at least four of seven predefined targets:

1. Blood pressure <140/90 mmHg
2. Normal weight or ≥5% weight loss if body mass index (BMI) >25 kg/m^2^
3. Waist circumference <88 cm (women) or <102 cm (men)
4. LDL cholesterol <55 mg/dL or ≥50% reduction from baseline
5. Smoking cessation
6. Adherence to a Mediterranean diet (PREDIMED score ≥7)
7. Physical activity ≥6000 steps/day or moderate/high activity on the International Physical Activity Questionnaire.

HbA1c was excluded from the composite to avoid denominator heterogeneity, as it applied only to patients with diabetes. The seven components reflect guideline-based secondary-prevention targets defined in absolute terms; two of them additionally allow a proportional-change criterion: weight (normal weight or a loss of at least 5% if BMI exceeded 25) and LDL cholesterol (<55 mg/dL or a reduction of at least 50% from baseline). Baseline LDL cholesterol was obtained from the in-hospital blood test performed during the index admission. This proportional criterion was applied because some patients cannot reach the absolute target within 8 weeks despite optimal adherence, such as those with very high baseline LDL recently started lipid-lowering therapy.

Secondary outcomes included:

- Functional capacity (METs, exercise test)
- Adherence to CR programme (≥70% session completion)
- Adherence to individualized exercise plan (3–5 sessions/week at prescribed intensity)
- Medication adherence (Morisky-Green questionnaire)
- Health-related quality of life (EQ-5D)
- Anxiety and depression [Hospital Anxiety and Depression Scale (HADS)]
- Patient satisfaction (visual analogue scale, 0–100)
- Cardiac rehospitalization, emergency visits, and revascularization procedures at 6 months.

Exercise adherence was assessed using the same predefined criteria in both study groups. Adherence to the CR programme was defined as attendance at ≥70% of scheduled sessions, and adherence to the prescribed exercise plan as completion of 3–5 sessions per week at the prescribed intensity. Attendance and participation were prospectively recorded by the supervising physiotherapist at each session, either remotely via synchronous videoconference (telerehabilitation group) or on-site (centre-based group).

Functional capacity was assessed at baseline and after the 8 week programme using a symptom-limited treadmill exercise stress test (Bruce protocol), expressed in METs; the baseline test was used to confirm eligibility, stratify exercise-related risk, and individualize aerobic exercise prescription.

### Follow-up

Participants were assessed at baseline, at the end of the 8 week programme (visit 2), and at 6 months (visit 3). Patient satisfaction was assessed at the end of the programme using two 0–100 visual analogue scale (VAS) items (overall satisfaction and willingness to recommend the programme).

### Statistical analysis

The trial was designed as a non-inferiority study to detect whether telerehabilitation does not have inferior outcomes than centre-based rehabilitation. Assuming a responder rate of 80% in both groups and a clinically relevant margin (delta) of 20%, a sample size of 116 patients (58 per group) was calculated with 80% power and an alpha level of 0.05, allowing for 10% dropout.

Descriptive statistics were calculated for all study variables. Continuous variables are presented as mean ± standard deviation (SD) or median (25th–75th percentile), as appropriate. Categorical variables are expressed as counts and percentages. Missing data are reported for each group where applicable.

The primary analysis was performed according to the Full Analysis Set, consistent with the intention-to-treat principle. In accordance with recommendations for non-inferiority trials, a per-protocol (PP) analysis was also conducted as a sensitivity analysis. Non-inferiority was considered demonstrated only if both analyses supported the same conclusion.

Comparisons of categorical variables were performed using the χ^2^ test or Fisher’s exact test, as appropriate. Continuous variables were compared using the independent samples Student’s t-test or analysis of variance (ANOVA) for comparisons involving more than two groups. When normality assumptions were not met, the Mann–Whitney U test was applied. Longitudinal within-group comparisons were assessed using the paired Student’s t-test.

The primary non-inferiority analysis was conducted using a one-sided α level of 0.025, corresponding to a two-sided 95% confidence interval. Secondary endpoints were analysed using two-sided tests with a significance level of 0.05.

All analyses were performed in accordance with the principles outlined in the ICH E9 guideline and Good Clinical Practice standards, using SAS software (version 9.4 or later; SAS Institute Inc., Cary, NC, USA).

### Ethics

The study protocol was approved by the Clinical Research Ethics Committee of the hospital (version 2.0, dated 20 October 2021), and all participants provided written informed consent. The trial was conducted in accordance with the Declaration of Helsinki and the European General Data Protection Regulation.

## Results

### Participant flow and baseline characteristics

A total of 164 patients were assessed for eligibility, and 129 were enrolled and randomized (65 to telerehabilitation and 64 to centre-based CR). Of these, 116 (89.9%) completed the 8 week intervention, and 109 (84.5%) completed the final follow-up visit. The reasons for attrition were similar in both groups and included adverse cardiac events, logistical conflicts, and loss to follow-up (*Figure 1*).

**Figure 1:**
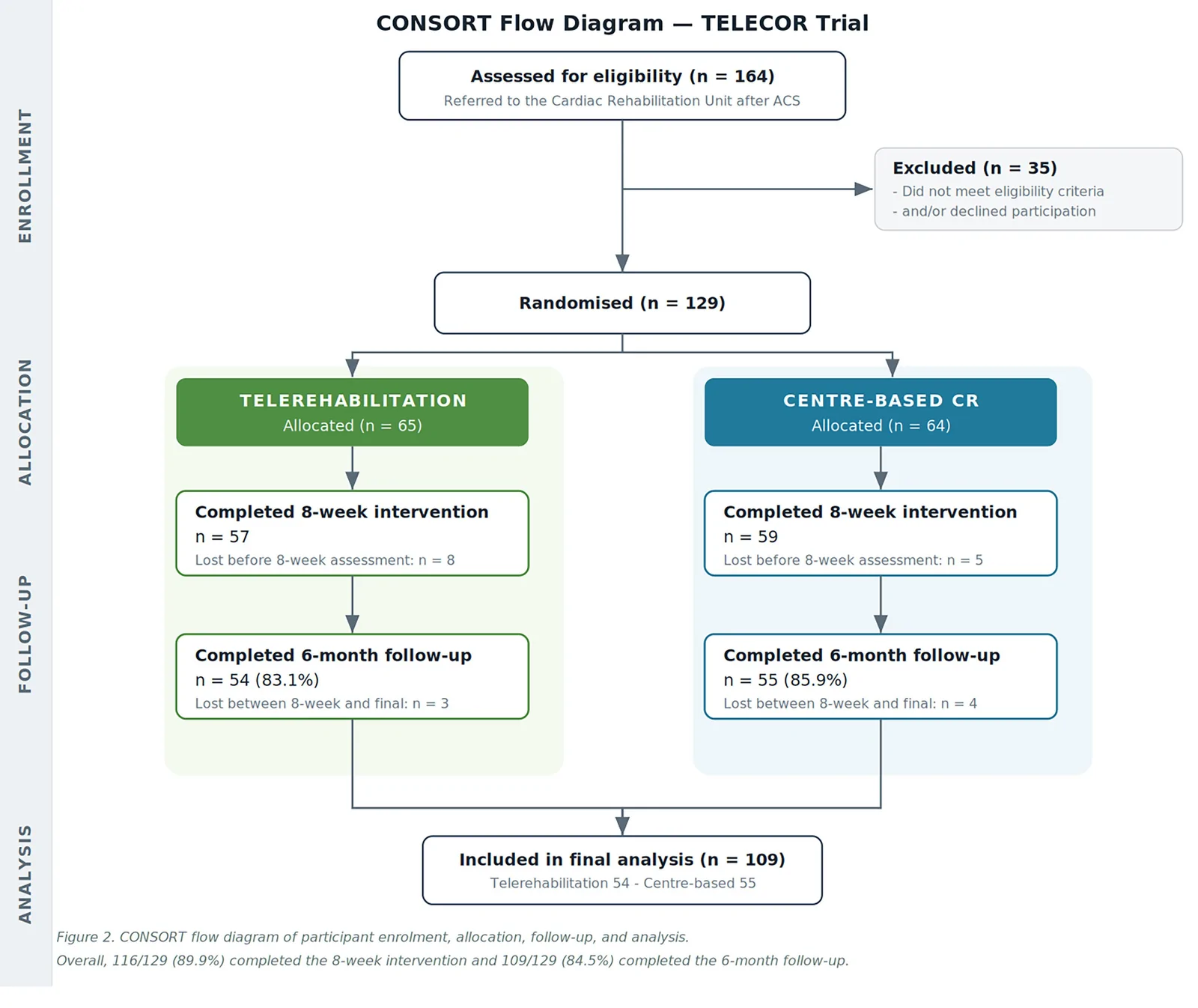
CONSORT flow diagram of participant enrolment, allocation, follow-up, and analysis. Overall, 116/129 (89.9%) completed the 8 week intervention and 109/129 (84.5%) completed the 6 month follow-up.

As shown in *Table 1*, both groups were well balanced at baseline across demographic, clinical, and cardiovascular risk parameters. Mean age was 57.6 years, 86.8% were male, and 82.2% had ST-segment elevation myocardial infarction. Left ventricular ejection fraction was mildly reduced (35 to <45%) in only a small, comparable proportion of participants (telerehabilitation 7.8% and centre-based 9.4%). No significant differences were observed in blood pressure, BMI, waist circumference, LDL cholesterol, smoking status, diet adherence, or physical activity level (*P* > 0.5 for all comparisons) (*Table 1*).

**Table 1:** Baseline comparability of risk factor profiles between groups

Variable	Telerehabilitation	Center-based CR	*P*-value
Age (years)	56.58 ± 9.09	58.64 ± 9.05	0.2
Male sex (%)	86.20	87.50	0.82
STEMI diagnosis (%)	89.20	75	0.03
LVEF (%)	54 ± 7.5	54.22 ± 8.	0.87
LVEF < 45% (%)	7.8	9,4	1.00
Hypertension (%)	36.9	45.3	0.33
Diabetes (%)	20	21.9	0.79
Dyslipidaemia (%)	69.2	62.5	0.42
BMI > 25 kg/m^2^ (%)	76.2	81	0.86
Waist circumference > target (%)	43.5	64.5	0.06
LDL ≥ 55 mg/dL (%)	95.2	93.5	0.69
Current smoker (%)	56.9	50	0.43
Low Mediterranean diet adherence Predimed < 7 (%)	10.7	19.3	0.2
Physical inactivity (IPAQ low or <6000 steps/day) (%)	21.8	23.6	0.82

BMI, body mass index; IPAQ, International Physical Activity Questionnaire; LVEF: left ventricular ejection fraction; STEMI: ST elevation myocardial infarction.

### Primary outcome

At 8 weeks, 82.8% of participants achieved the composite endpoint of meeting at least four of seven predefined cardiovascular risk and lifestyle goals. The proportion was slightly higher in the telerehabilitation group (86%) compared to centre-based CR group (79.7%), although the difference was not statistically significant (*P* = 0.37).

This improvement was sustained at the final follow-up (visit 3), with 87% of telerehabilitation patients and 80.4% of centre-based patients maintaining target adherence (*P* = 0.34). At the 6 month final visit, the proportion of patients meeting individual cardiovascular risk factor targets was similar between the telerehabilitation and centre-based CR groups. No statistically significant differences were observed for any individual target, supporting the non-inferiority of telerehabilitation in controlling cardiovascular risk factors.

*Figure 2* shows the breakdown of individual criteria met at the final visit. Control of blood pressure, smoking abstinence, adherence to a diet healthy and an adequate level of physical activity were the most frequently achieved goals (>80% in both groups), while waist circumference and body weight targets were less frequently met (<60%).

**Figure 2:**
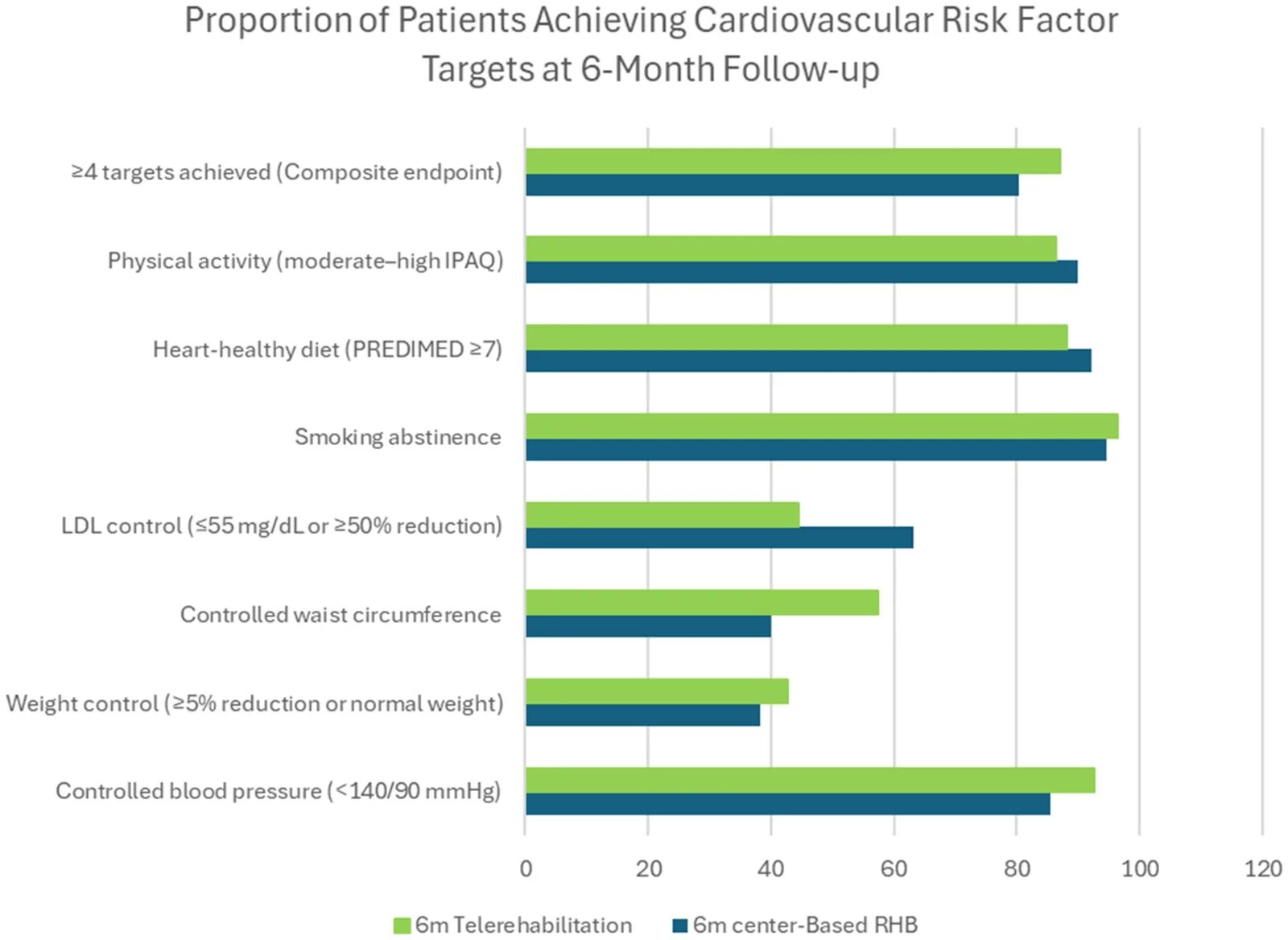
Proportion of patients meeting each individual cardiovascular risk factor target at the final visit.

Moreover, *Figure 3* illustrates the evolution of the composite endpoint across study visits.

**Figure 3:**
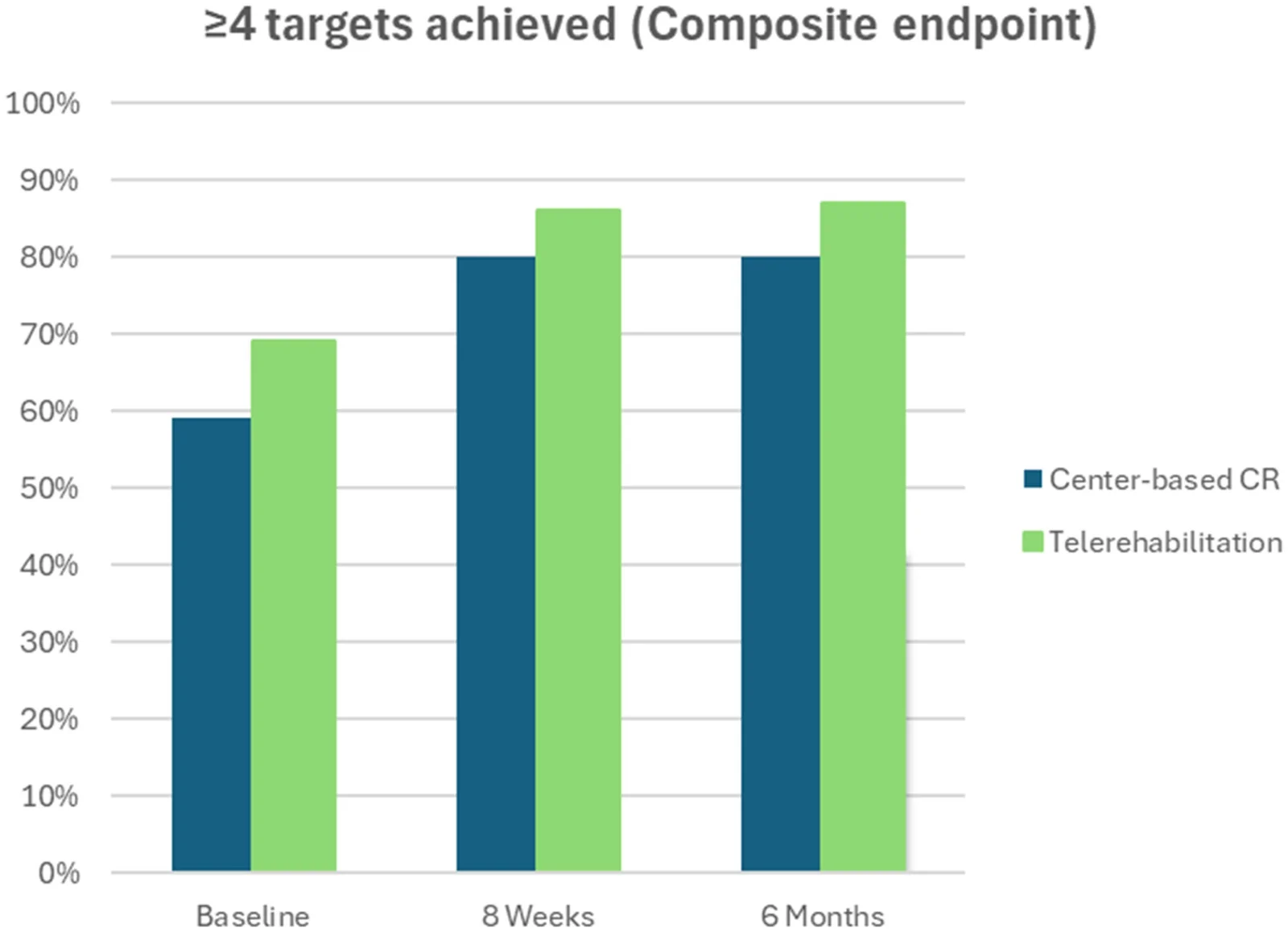
Evolution of the composite endpoint across study visits.

### Secondary outcomes

Both groups showed significant improvement in functional capacity. Telerehabilitation increased from 6.81 to 7.67 METs (*P* = 0.02), and centre-based CR from 6.08 to 7.53 METs (*P* < 0.0001). The between-group difference at follow-up was not significant (*P* = 0.67). Baseline functional capacity was significantly lower in the centre-based group (6.08 vs. 6.81 METs; *P* = 0.048), which provided greater room for improvement; both groups reached a comparable final capacity.

Programme adherence (≥70% session completion) was 88.3% in telerehabilitation vs. 84.8% in centre-based (*P* = 0.11). Exercise plan adherence was high in both groups. Medication adherence was 100% across all visits in both arms. Quality of life (EQ-5D index) remained stable and high, while EQ-5D VAS improved more in the telerehabilitation group within-group change +7.2 vs. +0.7 points; the higher value in the telerehabilitation group at 8 weeks was significant in the cross-sectional comparison, *P* = 0.034). HADS scores improved modestly in both groups, with no between-group differences (*Table 2*).

**Table 2:** Within-group changes over time

Outcome	Telerehab: Baseline → 8w (*P*)	Telerehab: 8w → Final (6 months)	Center-based: Baseline → 8w (*P*)	Center-based: 8w → Final (6 months)
METs	6.81 → 7.67 (*P* = 0.02)	7.67 → NA	6.08 → 7.53 (*P* < 0.0001)	7.53 → NA
EQ-5D index	0.91 → 0.91 (NS)	0.91 → 0.90 (NS)	0.91 → 0.92 (NS)	0.92 → 0.92 (NS)
EQ-5D VAS	67.9 → 75.1 (*P* = 0.03)	75.1 → 74.2 (NS)	64.6 → 65.3 (NS)	65.3 → 70.9 (NS)
HADS score	10.95 → 8.94 (NS)	8.94 → 9.32 (NS)	10.12 → 8.98 (NS)	8.98 → 9.08 (NS)

EQ-5D VAS, EQ-5D visual analogue scale score; HADS, Hospital Anxiety and Depression Scale; METs, metabolic equivalent of task; NA, not assessed; NS, not significant.

At 8 weeks, the proportion of participants free of any cardiac event was high and comparable between groups (98.2% telerehabilitation vs. 94.9% centre-based; *P* = 0.37); at 6 months, the proportions were 92.6% and 96.3%, respectively (*P* = 0.71), with no significant between-group differences. Satisfaction with the programme was high (91.73 ± 11.48 on a 0–100 VAS), and willingness to recommend the programme was also high (mean recommendation score 98.58 ± 4.55 on the same scale). A follow-up rate of 84.5% at 6 months strengthens the validity of our findings by minimizing attrition bias and ensuring sufficient power to assess sustained outcomes.

## Discussion

### Summary of main findings

TELECOR makes four specific contributions: it is, to our knowledge, the first pragmatic randomized trial to compare telerehabilitation with centre-based cardiac rehabilitation using a multidimensional composite endpoint aligned with ESC and AHA/ACC guideline targets; it was conducted entirely within a publicly funded tertiary hospital and integrated into the routine cardiac rehabilitation pathway; it focused on patients with recent ACS (within 2 months); and follow-up was maintained at 6 months in over 84% of participants.

The TELECOR study suggests that a structured telerehabilitation programme integrated into a public tertiary care hospital was non-inferior to standard centre-based cardiac rehabilitation in achieving guideline-based secondary prevention goals after ACS. A large proportion of participants in both groups achieved at least four of the seven predefined cardiovascular risk factors and healthy lifestyle targets. Functional capacity and patient satisfaction improved similarly in both groups. Although the study was not powered to evaluate clinical events, the observed event rates are consistent with the short-term safety of telerehabilitation.

### Comparison with previous studies

Our results are consistent with previous systematic reviews and trials, including the 2017 Cochrane review by Anderson *et al*., which found no significant differences between home-based and centre-based CR in terms of mortality, exercise capacity, health-related quality of life, and programme completion.^14,15^ Showing that home-based and centre-based CR yield similar gains in peak VO_2_ and health outcomes. Likewise, large trials and statements have reported equivalent improvements in exercise capacity with tele-supervised programmes (≈1 MET).^16,17^ Adherence rates in telerehabilitation randomized controlled trials (RCTs) have ranged from 70% to 90%,^3,4^ consistent with the 88.3% session completion observed in TELECOR. Patient satisfaction and recommendation rates in our study (>90/100) also mirror high acceptability reported in prior tele-CR evaluations.^18^

TELECOR complements these findings by incorporating a multidimensional composite endpoint for cardiovascular risk factor control—an aspect not systematically evaluated in prior studies. Moreover, our real-world implementation in a publicly funded system adds a pragmatic dimension often lacking in earlier RCTs.

Recent trials, such as those by Dalli Peydró *et al*.^10^ and Owen *et al*.,^18^ similarly report that telerehabilitation achieves comparable improvements in physical performance and satisfaction. Our results reinforce those findings, with patient satisfaction exceeding 91 points on a 0–100 scale and a mean recommendation score above 98 on the 0–100 scale. This aligns with evidence from Thomas *et al*.,^8^ who emphasized patient-centred benefits of remote models.

### Strengths and limitations

A major strength of this study is its pragmatic design and integration into routine care pathways in a public tertiary hospital, enhancing external validity. The composite endpoint, aligned with ESC and AHA/ACC guideline targets,^1,2^ provides a more holistic measure of secondary prevention success than traditional single-metric trials. High follow-up rates (84.5% at 6 months) bolster the validity of our findings. The use of a guideline-derived composite endpoint provides a clinically meaningful measure of success beyond isolated physiological markers. The trial also benefited from high adherence and retention rates.

However, limitations should be acknowledged. First, key variables such as physical activity, diet, and satisfaction were assessed through self-report and, in the case of satisfaction, with a single-item 0–100 scale rather than a fully validated satisfaction questionnaire, which may introduce bias. Second, the sample size, while sufficient for detecting non-inferiority in the primary outcome, may not have been powered to detect more subtle differences in secondary outcomes such as psychological status or adverse events. Third, the single-centre design may limit generalizability, although its real-world setting improves applicability to similar health systems. Fourth, screening failures were not systematically categorized, preventing detailed reporting of the reasons for non-participation.

### Clinical and research implications

TELECOR shows that structured telerehabilitation can be implemented at scale in public health systems, achieving equivalent secondary prevention results to centre-based CR. These findings support the integration of structured telerehabilitation into routine secondary prevention programmes. Digital delivery models can effectively complement or substitute traditional CR when integrated within clinical workflows and tailored to patients’ capabilities and contexts. However, not all patients may be suitable for remote interventions—particularly those with cognitive impairment, low digital literacy, or lack of access to technology.^18,19^ Conversely, centre-based CR faces persistent access barriers such as travel, rigid scheduling, and institutional limitations.^18^ Future research should explore hybrid or adaptive strategies, assess long-term outcomes, and investigate implementation frameworks that maximize reach and equity.

The high adherence and satisfaction suggest tele-delivery may overcome traditional barriers such as travel distance and scheduling conflicts.^9^ Future research should evaluate long-term cardiovascular events, cost-effectiveness, and equity of access across diverse populations. Integration of digital CR into national guidelines and reimbursement frameworks could substantially expand CR coverage and improve population-level outcomes.

## Supplementary Material

oeag142_Supplementary_Data

## Data Availability

The data supporting the findings of this study are available from the corresponding author upon reasonable request.
