# Supplementary material for: Telerehabilitation vs. centre-based cardiac rehabilitation after acute coronary syndrome: results of the pragmatic TELECOR randomized trial
Source: Eur Heart J Open. 2026 Sep 24;6(5):oeag142. doi: 10.1093/ehjopen/oeag142 (PMC13675247; doi:10.1093/ehjopen/oeag142)
Supplement: Supplementary file 1 [file oeag142_supplementary_data.docx]

# **Supplementry Appendix S1**

# **Table S1. Consensus on Exercise Reporting Template (CERT**

| **CERT item** | **Description** |
| --- | --- |
| **1. Exercise equipment** | The centre-based rehabilitation group used treadmills, cycle ergometers and TheraBand® elastic resistance bands. Participants allocated to the telerehabilitation group received the prescribed TheraBand® resistance bands (blue, black, silver or gold) during the initial face-to-face visit. Heart rate was monitored using Fitbit Inspire 2 devices integrated into the Vitalera platform in the telerehabilitation group and using custo belt chest-worn heart rate monitors (custo med GmbH, Ottobrunn, Germany) during supervised exercise sessions in the centre-based rehabilitation group. |
| **2. Exercise providers** | Exercise sessions were supervised by physiotherapists specialised in cardiac rehabilitation. Overall clinical supervision of both intervention groups was provided by a rehabilitation physician specialised in cardiac rehabilitation, who was responsible for clinical decision-making and management of any exercise-related or medical issues throughout the programme. |
| **3. Individual or group exercise** | Exercise sessions were delivered in groups of 4–6 participants in both intervention groups. Centre-based rehabilitation consisted of face-to-face group sessions, whereas telerehabilitation was delivered through synchronous group videoconference sessions using a secure telemedicine platform. |
| **4. Supervision** | All exercise sessions were supervised in real time by the physiotherapists, who monitored symptoms, exercise technique, heart rate, perceived exertion and adherence throughout each session |
| **5. Adherence assessment** | Attendance was recorded at every supervised session. Programme adherence was defined as attendance at ≥70% of scheduled sessions. Exercise adherence was defined as completing 3–5 exercise sessions per week at the prescribed intensity and was prospectively recorded using the same standardised exercise activity log in both intervention groups. |
| **6. Motivation strategies** | Participants received continuous verbal feedback during each exercise session. Progress and adherence were reviewed throughout the programme, and communication with the rehabilitation team was available through the Vitalera platform. |
| **7a. Progression rules** | Aerobic exercise intensity was prescribed according to the baseline symptom-limited treadmill exercise test (Bruce protocol). Participants achieving maximal heart rate exercised at 75% of maximal heart rate during weeks 1–4 and progressed to 85% during weeks 5–8, provided exercise was well tolerated. Participants who did not achieve maximal heart rate followed the same progression using heart rate reserve (Karvonen method). Resistance training was progressed according to weekly reassessment of the 10-repetition maximum (10-RM). |
| **7b. Progression implementation** | Progression criteria were identical in both intervention groups. Physiotherapists continuously monitored heart rate, symptoms and perceived exertion. Aerobic intensity increased from 75% to 85% after four weeks when appropriate. Resistance was adjusted weekly according to repeated 10-RM assessments |
| **8. Exercise session** | Each supervised session lasted approximately 60 minutes and consisted of 20–25 minutes of resistance training, 25–30 minutes of aerobic exercise and 5–10 minutes of cool-down. |
| **8a. Aerobic exercise** | Aerobic exercise was performed at the prescribed target intensity. Centre-based participants exercised on treadmills or cycle ergometers. Telerehabilitation participants completed a supervised home-based circuit including marching in place, jogging in place, side stepping, step-touch movements, alternating knee lifts, hamstring curls, low-impact jumping jacks, boxing movements and, when appropriate, stair climbing. Exercise pace was continuously adjusted to maintain the target heart rate. |
| **8b. Resistance exercise** | Resistance training targeted the major upper- and lower-limb muscle groups using TheraBand® elastic resistance bands. Exercises included shoulder flexion and abduction, elbow flexion and extension, seated row, squats, sit-to-stand, hip abduction and extension, knee extension and plantar flexion. Participants completed 2–3 sets of 10 repetitions with approximately 1 minute of rest between sets. Intensity was prescribed according to the baseline 10-RM and monitored using the OMNI-Resistance Exercise Scale (OMNI-RES; target 4–6). |
| **9. Home-based or non-exercise setting** | The telerehabilitation programme was delivered in participants' homes through supervised synchronous group videoconference sessions. The centre-based programme was delivered in the Cardiac Rehabilitation Unit of a tertiary public hospital. |
| **10. Non-exercise components** | Both intervention groups received the same multidisciplinary educational programme covering cardiovascular risk factor management, smoking cessation, Mediterranean diet counselling, medication adherence and healthy lifestyle promotion. |
| **11. Adverse event monitoring** | Participants were monitored throughout all supervised sessions. Exercise-related and medical incidents were assessed by the supervising physiotherapists and referred to the rehabilitation physician when appropriate. Cardiac events were prospectively recorded during the intervention and throughout the 6-month follow-up. |
| **12. Exercise location** | Centre-based rehabilitation was delivered in the Cardiac Rehabilitation Unit of a tertiary public hospital. Telerehabilitation was delivered in participants' homes through a secure telemedicine platform providing synchronous group supervision. |
| **13. Tailoring** | Exercise prescription was individualised according to baseline functional capacity. Aerobic intensity and resistance training were individually prescribed and progressively adjusted according to predefined criteria. |
| **14. Starting level** | Initial aerobic intensity was prescribed at 75% of maximal heart rate or, when appropriate, 75% of heart rate reserve (Karvonen method). Initial resistance was prescribed according to the baseline 10-RM using TheraBand® elastic resistance bands. |
| **15. Fidelity assessment** | Attendance, exercise completion, adherence, progression and heart-rate monitoring were continuously assessed throughout the intervention. Fitbit Inspire 2 devices supported monitoring in the telerehabilitation group, whereas custo belt chest-worn heart rate monitors were used during supervised sessions in the centre-based group. |
| **16. Fidelity achieved** | The intervention was delivered as planned in both groups under continuous real-time supervision by physiotherapists specialised in cardiac rehabilitation. Exercise prescription, progression criteria and execution were consistently implemented. The multidisciplinary rehabilitation programme was identical in both groups, differing only in the exercise setting and aerobic exercise modality. |

|  |  |
| --- | --- |
|  |  |
|  |  |
|  |  |
|  | . |
|  |  |
|  |  |
|  |  |
|  | . |
|  |  |
|  |  |
|  |  |
|  |  |
|  |  |
|  |  |
|  |  |
|  |  |
|  |  |
|  |  |
|  |  |
